# Prognostic significance of Ki67 in Chinese women diagnosed with ER^+^/HER2^−^ breast cancers by the 2015 St. Gallen consensus classification

**DOI:** 10.1186/s12885-016-3021-7

**Published:** 2017-01-06

**Authors:** Yue Hu, Ran Gu, Jinghua Zhao, Yaping Yang, Fengtao Liu, Liang Jin, Kai Chen, Haixia Jia, Hongli Wang, Qiang Liu, Fengxi Su, Weijuan Jia

**Affiliations:** 1 Guangdong Provincial Key Laboratory of Malignant Tumor Epigenetics and Gene Regulation, Sun Yat-Sen Memorial Hospital, Sun Yat-Sen University, Guangzhou, 510120 People’s Republic of China; 2 Breast Tumor Center, Sun Yat-Sen Memorial Hospital, Sun Yat-Sen University, Guangzhou, 510288 People’s Republic of China; 3 Department of Breast Surgery, Second Affiliated Hospital of Guangzhou Medical University, Guangzhou, 510260 People’s Republic of China

**Keywords:** ER^+^/HER2^−^ breast cancer, Ki67-labeling index, Prognosis

## Abstract

**Background:**

This study evaluated the distribution pattern of the Ki67-labeling index (LI) among patients at a Chinese breast cancer center, and analyzed its prognostic significance in the 2015 St Gallen consensus breast cancer classification, estrogen receptor-positive and human epidermal growth factor receptor 2-negative(ER^+^/HER2^−^)subtype.

**Methods:**

We classified 939 women with ER^+^/HER2^−^ breast cancer into three groups by Ki67-LI levels, and followed their clinicopathologic characteristics and prognoses.

**Results:**

In the 939 eligible subjects, 342 had Ki67-LI ≤10% (Ki67^Low^), 281 had Ki67-LI between 10 and 30% (Ki67^Medium^), and 316 had Ki67-LI ≥30% (Ki67^High^). Although the Ki67^High^ group had less favorable clinicopathologic factors, the Ki67^Medium^ group’s factors varied considerably. Kaplan-Meier estimates showed that disease-free survival(DFS) for the Ki67^Medium^ group was significantly shorter than the Ki67^Low^ group but longer than the Ki67^High^ group. Ki67-LI had independent prognostic significance in multivariate analysis. Other diagnostic factors, including tumor size >2 cm, positive lymph nodes, and grade III disease, were significantly associated with poorer disease-free survival only in the Ki67^Medium^ group.

**Conclusions:**

For patients with ER^+^/HER2^−^ breast cancer, we confirmed three distinct risk patterns by Ki67-LI levels according to the 2015 St Gallen consensus. For patients with clearly low or high Ki67-LI, straightforward clinical decisions could be offered, but for patients with intermediate Ki67-LI, other factors might provide valuable information.

**Electronic supplementary material:**

The online version of this article (doi:10.1186/s12885-016-3021-7) contains supplementary material, which is available to authorized users.

## Background

Breast cancer (BC) is a molecularly heterogeneous disease that includes at least four intrinsic subtypes with different features and prognoses [1–5]. Among estrogen receptor-positive and human epidermal growth factor receptor 2-negative (ER^+^/HER2^−^) BCs, gene expression-based assays showed that they can be divided into at least two distinct subgroups—luminal A and luminal B [4]. Although the luminal B subset has higher proliferation marker expression and worse prognosis [6], clinicopathological subtyping criteria of the two luminal groups keep changing in the literature.

The 2011 St Gallen consensus panel [7] recommended a cut-off of 14% for the Ki67-labeling index (LI) as the threshold between luminal A and B subtypes. The 2013 St Gallen consensus panel added another immunohistochemical (IHC) surrogate marker—progesterone receptor (PgR)—and increased the Ki67-LI cut-off to 20% [8]. However, at the latest 2015 St Gallen International Breast Cancer Conference, the panel recommended Ki67-LI should be interpreted upon local laboratory values, and ER^+^/HER2^−^ BCs could not be classified as two distinctive groups by IHC surrogate markers, as they belong to a spectrum of disease [9].

This study evaluated Ki67-LI distribution in a Chinese BC treatment center and analyzed its prognostic significance in the 2015 St Gallen consensus of ER^+^/HER2^−^ BCs.

## Methods

### Patients and clinical data collection

We searched breast tumor registries of the 2063 patients who had been treated for BC at the Sun Yat-Sen Memorial Hospital from March 2005 to December 2012, and for whom Ki67-LI information was available. Our hospital is equipped with one of the most comprehensive breast centers in China, and we are highly recognized by patients from all over the country. We excluded patients with non-invasive BC, ER-negative or HER2-positive disease, more than three involved lymph nodes, T4 lesions, male patients, and those with distant metastasis at first diagnosis. Finally, we included 939 patients with early invasive ER^+^/HER2^−^ BCs.

Of these 939 participants, 372 (39.6%) received modified radical mastectomies and 567 (60.4%) underwent breast-conserving surgery (BCS, all with negative margins). All patients received sentinel lymph node biopsy (SLNB), of whom SLNB positive went on to axillary lymph node dissection(ALND). After surgery, 776 (82.6%) patients received adjuvant chemotherapy. The regimens mainly consisted of three types: standard cyclophosphamide, methotrexate, and 5-fluorouracil, anthracycline-based, or combined anthracycline and taxane (termed taxane-based regimen). They each received four to eight chemotherapy cycles. The main indications for radiotherapy included positive lymph nodes; primary tumor >5 cm; or BCS. In our study, chemotherapy indications were based on the National Comprehensive Cancer Network (NCCN) guidelines.

Patients were followed according to clinical protocols. The evaluated endpoint was DFS, which was defined as the interval from first diagnosis until diagnosis of local or regional BC recurrence, contralateral BC, distant metastasis, or death from BC. Patients known to be alive without recurrent disease or lost to follow-up at the time of analysis were screened at the time of their last follow-up. For those who attended no further clinical visits at our institute, essential follow-up information was collected by telephone. The cut-off date for the results presented here was February 22, 2016. This study was approved by the Ethics Committee of Sun Yat-Sen Memorial Hospital, Sun Yat-Sen University.

### Laboratory methods and group definition

IHC staining for ER, PgR, HER2 protein, and Ki67 antigen was performed on core biopsies or surgical specimens. All specimens were fixed with 10% neutral phosphate-buffered formalin and embedded in paraffin. Samples were considered ER^+^ if more than 10% nuclei were stained, which is the usual cut-off in larger Chinese hospitals [10]. Tumors were considered HER2^+^ if they received a score of three by IHC or if they were two by IHC but had amplified HER2 genes (ratio 2.0) based on fluorescence in situ hybridization (FISH). The MIB-1 clone antibody (1:100, DAKO) was used for Ki67 IHC staining; the Ki67-LI was the percentage of positively stained cancer cells (regardless of staining intensity).

Based on the 2015 St Gallen consensus standard, and the median Ki67-LI value(20%) of patients in this study, the 939 women were classified into three groups by cut-off points 10 and 30% [9]. Patients whose Ki67-LI was 30% or above were considered clearly high (Ki67^High^); those whose Ki67-LI was 10% or less were clearly low (Ki67^Low^), and those whose Ki67-LI was 10–30% were considered intermediate (Ki67^Medium^).

### Statistical analysis

Statistical comparisons of clinicopathological characteristics within the three groups were calculated by chi-square and rank-sum tests. Kaplan-Meier survival curves were calculated; log-rank test was adopted to compare DFS within groups. Multivariate DFS analysis used the Cox proportional hazards model. SPSS 19.0 (SPSS Inc., Chicago, IL, USA) was used for statistical analyses. All *P* values were two-tailed; *P* ≤ 0.05 was considered significant.

## Results

For the 939 patients who were eligible for inclusion in this study, the median follow-up period was 64.7 months (range:4.3–120.7 months), and the mean follow-up period was 67.2 months (SD:23.4 months). The Ki67-LIs were intensively clustered at values ending with 5 or 0 (829, 88.3%). The median value of Ki67-LI was 20% for these 939 cases. Among the 939 patients, 342 cases had Ki67-LI ≤ 10%, 281 had Ki67-LI between 10 and 30%, and 316 had Ki67-LI ≥ 30%. If all 2063 cases with Ki67-LI data were included for analysis regardless of subtype, median value increased to 25%. Ki67-LIs of this cohort also intensively clustered at values ending with 5 or 0 (1886, 91.4%).

Table 1 shows the clinicopathological characteristics in each group and indicates significant differences in age distribution, menopausal status, histology, tumor size, node involvement, grade, and PgR status. No significant differences within groups were seen for median age or lymphovascular invasion (LVI) status. The proportion of early-onset BC (age <35 years) or PgR-negative/low was higher in the Ki67^High^ group than other groups. Tumors <2 cm or without lymph node involvement were more commonly observed in the Ki67^Low^ group. Almost all tumors in the Ki67^Low^ group were grade I/II, whereas more than half the cases in the Ki67^High^ group were grade III. Nevertheless, the clinical features of the Ki67^Medium^ group were not remarkable, and for many cases, it was hard to differentiate them from the Ki67^Low^ or Ki67^High^ groups. The largest proportion of patients who received chemotherapy were from the Ki67^High^ group, and the largest proportion of patients who received radiotherapy were from the Ki67^Low^ group. The groups did not significantly differ by surgery type.

**Table 1:** Clinicopathologic Characteristics of three groups according to Ki67-LI level

Characteristic	Ki67 ≤ 10% *n* = 342	10% < Ki67 < 30% *n* = 281	Ki67 ≥ 30% *n* = 316	Total *N* = 939	*P* value
Associated events-no.(%)	10(2.9)	20(7.1)	49(15.5)	79(8.4)	
Age					
Median(IQT)-yr	49(23–86)	48(24–91)	47(22–84)	48(22–91)	0.166
Distribution-no.(%)					0.013
< 35	19(5.6)	15(5.3)	31(9.8)	65(6.9)	
35–50	160(46.8)	148(52.7)	163(51.6)	471(50.2)	
51–65	122(35.7)	93(33.1)	102(32.3)	317(33.8)	
> 65	41(12.0)	25(8.9)	20(6.3)	86(9.2)	
Menopausal status-no.(%)					0.011
Pre	171(50.0)	122(43.4)	176(55.7)	469(49.9)	
Post/Peri	171(50.0)	159(56.6)	140(44.3)	470(50.0)	
Histology-no.(%)					<0.001
IDC	273(79.8)	236(84.0)	297(94.0)	806(85.8)	
ILC	10(2.9)	10(3.6)	6(1.9)	26(2.8)	
Mucinous	25(7.3)	13(4.6)	4(1.3)	42(4.5)	
Other invasive histology	34(9.9)	22(7.8)	9(2.8)	65(6.9)	
pT-no./total no.(%)					<0.001
1	239/336(71.1)	181/276(65.6)	159/307(51.8)	579/919(63.0)	
2	91/336(27.1)	86/276(31.2)	139/307(45.3)	316/919(34.4)	
3	6/336(1.8)	9/276(3.3)	9/307(2.9)	24/919(2.6)	
Node-no.(%)					0.022
N0	249(72.8)	180(64.1)	202(63.9)	631(67.2)	
N1	93(27.2)	101(35.9)	114(36.1)	308(32.8)	
LVI-no.(%)					0.074
Positive	49(14.3)	59(21.0)	61(19.3)	169(18.0)	
Negative	293(85.7)	222(79.0)	255(80.7)	770(82.0)	
Grade-no./total no.(%)					<0.001
I/II	269/289(93.1)	189/241(78.4)	137/282(48.6)	595/812(73.3)	
III	20/289(6.9)	52/241(21.6)	145/282(51.4)	217/812(26.7)	
PgR-no.(%)					<0.001
< 20%	58(17.0)	55(19.6)	119(37.7)	232(24.7)	
≥ 20%	284(83.0)	226(80.4)	197(62.3)	707(75.3)	
Surgery-no.(%)					0.479
BCS	202(59.1)	178(63.3)	187(59.2)	567(60.4)	
Mastectomy	140(40.9)	103(36.7)	129(40.8)	372(39.6)	
Chemotherapy-no.(%)					<0.001
Yes	252(73.7)	234(83.3)	290(91.8)	776(82.6)	
No	90(26.3)	47(16.7)	26(8.2)	163(17.4)	
Radiotherapy-no.(%)					0.033
Yes	237(69.3)	217(77.2)	243(76.9)	697(74.2)	
No	105(30.7)	64(22.8)	73(23.1)	242(25.8)	

*IQT* interquartile range, *IDC* invasive ductal carcinoma, *ILC* invasive lobular carcinoma, *LVI* lymphovascular invasion, *PgR* progesterone receptor, *BCS* breast-conserving surgery

**Fig. 1:**
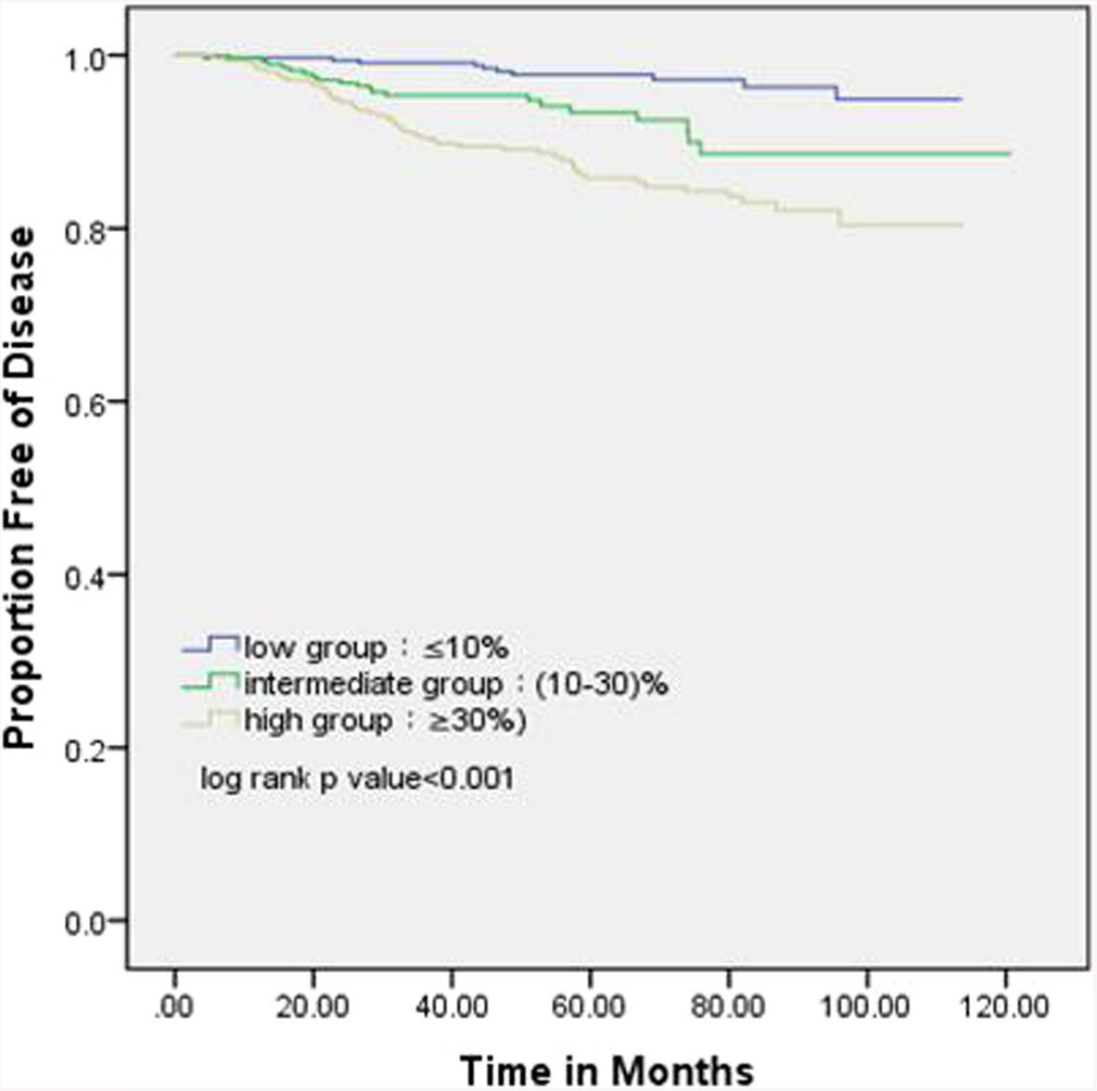
Disease-free survival according to Ki67-LI level group by Kaplan-Meier analysis

A total of 79 (8.4%) patients had BC-associated disease (Ki67^Low^: 10; Ki67^Medium^: 20; Ki67^High^: 49), including 12 local recurrences, seven regional nodal recurrences, three contralateral new BCs, 54 distant metastases, and three deaths from BC. The estimated 5-year DFS rate for the Ki67^Medium^ group (93%) was significantly less than for the Ki67^Low^ group (97%; log-rank *P* = 0.003), but significantly better than for the Ki67^High^ group (85%, log-rank *P* = 0.014; Fig. 1).

Our univariate analysis associated age younger than 35 years, tumor >2 cm, lymph node involvement, positive LVI status, grade III disease, higher Ki67-LI group, and surgical approach (mastectomy vs. BCS) with worse prognoses (Table 2). In multivariate analysis, the Ki67-LI groups were a significant independent predictor for DFS after adjusting for clinicopathological parameters and treatments (Table 2).

**Table 2:** Results of disease-free survival analysis by Cox proportional hazards model for the ER+/HER2− tumors, *n* = 939

	Univariate				Multivariate			
	HR	95%	CI	*P* value	HR	95%	CI	*P* value
Age (years)								
35–50 vs < 35	0.386	0.196	0.763	0.006	0.342	0.170	0.687	0.003
51–65 vs < 35	0.477	0.238	0.959	0.038	0.454	0.220	0.936	0.032
> 65 vs < 35	0.378	0.140	1.021	0.055	0.274	0.080	0.938	0.039
pT								
T2 vs T1	2.162	1.348	3.468	0.001	1.731	1.054	2.844	0.030
T3 vs T1	4.654	1.948	11.118	0.001	3.219	1.288	8.045	0.012
N								
N1 vs N0	1.826	1.173	2.844	0.008	1.797	1.007	3.207	0.047
LVI								
positive vs negative	1.831	1.099	3.048	0.020	1.332	0.776	2.284	0.298
Grade								
III vs I/II	2.890	1.808	4.619	<0.001	1.785	1.059	3.010	0.030
Ki67(%)								
10–30 vs ≤10	2.859	1.336	6.116	0.007	2.835	1.312	6.126	0.008
≥ 30 vs ≤10	5.394	2.732	10.651	<0.001	4.239	2.057	8.735	<0.001
PgR(%)								
< 20 vs ≥20	1.273	0.778	2.083	0.336	0.799	0.472	1.355	0.405
Surgery								
Mastectomy vs BCS	1.658	1.065	2.581	0.025	0.941	0.472	1.874	0.862
Chemotherapy								
No vs Yes	0.731	0.376	1.419	0.354	1.430	0.620	3.298	0.402
Radiotherapy								
No vs Yes	1.291	0.803	2.074	0.292	2.157	0.950	4.894	0.066

To refine prognostication for the Ki67^Medium^ group, we investigated the value of conventional clinical parameters. Interestingly, these factors, including tumor size >2 cm, lymph node status (N1 vs. N0), and grade III disease, had significant prognostic value in univariate and multivariate analyses of DFS for the Ki67^Medium^ group (Table 3), but not the Ki67^Low^or Ki67^High^ groups (Additional file 1: Table S1 and Additional file 2: Table S2).

**Table 3:** Results of disease-free survival analysis by Cox proportional hazards model for the Ki67^Medium^ group

	Univariate				Multivariate			
	HR	95%	CI	*P* value	HR	95%	CI	*P* value
Age (years)								
35–50 vs < 35	0.865	0.110	6.797	0.891	0.872	0.098	7.723	0.902
51–65 vs < 35	0.938	0.115	7.675	0.952	1.218	0.130	11.454	0.863
> 65 vs < 35	0.994	0.090	11.048	0.996	0.605	0.028	13.007	0.748
pT								
T2 vs T1	2.778	1.068	7.229	0.036	2.781	1.004	7.701	0.049
T3 vs T1	9.961	0.036	37.642	0.001	6.421	1.226	33.629	0.028
N								
N1 vs N0	3.087	1.255	7.589	0.014	4.572	1.500	13.933	0.008
LVI								
positive vs negative	2.476	0.979	6.263	0.048	1.434	0.517	3.977	0.488
Grade								
III vs I/II	4.437	1.828	10.770	0.001	4.468	1.650	12.104	0.003
PgR(%)								
< 20 vs ≥20	0.714	0.209	2.437	0.591	0.602	0.159	2.274	0.454
Surgery								
Mastectomy vs BCS	2.455	1.001	6.020	0.050	0.452	0.108	1.890	0.277
Chemotherapy								
No vs Yes	0.556	0.129	2.396	0.431	1.048	0.116	9.452	0.967
Radiotherapy								
No vs Yes	2.042	0.830	5.021	0.120	8.786	1.779	43.388	0.008

## Discussion

In this study, we investigated clinicopathologic characteristics and prognoses of the three risk levels among patients with ER^+^/HER2^−^ BCs, according to the most recent consensus from the 2015 St Gallen Conference, which focuses on local laboratory Ki67-LIs. To our knowledge, such an evaluation based on the 2015 St Gallen Breast Cancer Conference consensus has never been published before.

Ki67 is a nuclear protein expressed in all active phases of the cell cycle except resting phase G_0_ [11]. Therefore, it can be used as an alternative marker of proliferation by IHC assessment in many malignancies, including BC. Ki67 has been extensively evaluated in both research and clinical settings [12], and clearly offers robust prognostic and predictive information [13, 14]. The St Gallen International BC Conferences have recommended using Ki67-LI to tailor treatment in ER^+^/HER2^−^ BCs from 2009 to 2015 [7–9, 15]. However, our understanding of Ki67 has expanded, including its clinical value and optimal cut-off point. The 2009 St Gallen consensus suggested Ki67-LI as a proliferation marker in choosing appropriate systemic treatment, categorizing it by three levels: low (≤15%), intermediate (16–30%), or high (>30%) [15]. After comparing Ki67-LI with PAM50 intrinsic subtyping, Cheang et al. suggested a cut-off point of 14% to distinguish luminal B from luminal A subtype in ER^+^/HER2^−^ BCs [16]. The 2011 St Gallen Conference then endorsed a 14% cut-off point between luminal A and B tumors [7]. Subtyping can be used to shape decisions about the use of cytotoxic chemotherapy for luminal BCs [7, 8]. Luminal A disease generally requires only endocrine therapy, whereas chemotherapy is usually indicated for luminal B diseases. In 2013, the cut-off point was increased to 20% by the St Gallen panel [8]. Meta-analysis also showed diverse cut-off points of Ki67-LI ranging from 3.5 to 34% with various definitions [13]. A recent meta-analysis of more than 60,000 patients confirmed the prognostic value of Ki67-LI, and found 25% to be an optimal cut-off point [17].

At the most recent St Gallen Conference, in 2015, the panel recognized the controversy of using Ki67-LI by IHC assessment for clinical decisions [9]. The previous efforts of finding a universal optimal cut-off point of Ki67 might have been in vain, as Ki67 presents as a continuum. Clinicians should therefore be cautious when using a single cut-off point to dichotomize Ki67 scores [6, 18]. Particularly, reproducibility across laboratories has proven to be unacceptably poor, which is the major obstacle for its clinical use [19]. Because of this, the American Society of Clinical Oncology (ASCO) and NCCN do not currently recommend Ki67 as a routine required marker in clinical practice ([20], https://www.nccn.org/professionals/physician_gls/pdf/breast.pdf). With the goal of harmonizing methodology, the International Ki67 in Breast Cancer Working Group proposed guidelines for Ki67 assessment [12]. However, the effect on actual practice is less than optimal [21, 22]. Studies [21] and new image analyses [23] that aim to increase concordance in Ki67 scoring are still under development.

The 2015 St Gallen consensus panel recommended interpreting Ki67-LI with local laboratory values [9], which was also suggested by the European Society for Medical Oncology (ESMO) clinical practice guidelines [24]. In our study, median Ki67-LI was 25% for all tumors and 20% for the ER^+^/HER2^−^ subset. The two values reported by Cserni study were 17 and 14%, respectively [22]. Although the numerical gaps between these two centers were only 8 and 6% for two different patient sets, the numerous patients within this range should not be ignored. Of the 939 ER^+^/HER2^−^ cases in our study, 214 (22.8%) had Ki67-LIs between 14 and 20%. Our Ki67-LI values also intensively clustered at those ending with 5 or 0 (91.4% for all tumors and 88.3% for ER^+^/HER2^−^ subset), as did those in the Cserni study [22]. This finding reflects a common practice of pathologists. In our 939 cases of ER^+^/HER2^−^ tumors, *none* of the Ki67-LIs were valued at 14%, and only four cases at 12% or 13%, but 103 patients—11%—had Ki67-LIs of 15%. In light of this pattern, the widely used 14% cut-off point recommended by the 2011 St Gallen Conference seems unreasonable.

When ER^+^/HER2^−^ cases were stratified into three levels by median Ki67-LI value 20%, the Ki67^High^ group (≥30%) had less-favorable clinicopathologic characteristics, including the highest percentages of patients younger than 35 years, large tumors, high histology grades, and low or negative PgR. The Ki67^Low^ group (≤10%) had some favorable features, and features of the Ki67^Medium^ group (10–30%) were between the other two groups. These findings confirmed the validity of these Ki67 groupings from a clinicopathologic perspective. The three groups differed significantly in DFS (*P* < 0.001), with the Ki67^Low^ group having the longest DFS and the Ki67^High^ group the shortest.

To refine prognostication within the Ki67^Medium^ group, we examined other conventional diagnostic factors. We found that tumors larger than 2 cm, lymph node involvement, and grade III disease were all associated with poorer prognoses in the Ki67^Medium^ group after adjusting for treatments; however, these parameters had no prognostic significance in Ki67^High^ and Ki67^Low^ groups. It is difficult to standardize scores for patients with Ki67^Medium^ LIs, as they suffer from the highest variability [25, 26]. Accordingly, when making clinical decisions about the inclusion of cytotoxic chemotherapy for ER^+^/HER2^−^ BCs with intermediate Ki67-LI, other conventional parameters may provide more valuable information rather than Ki67-LI, or, alternatively, multigene assays [26]. However, multigene assays are not readily available worldwide owing to high cost and technical requirements. The prognosis significance of chemotherapy was not shown in analyses for the Ki67^Medium^ group. This result may be due to the low percentage of patients without chemotherapy. Further researches are needed to study the prognosis significance of treatments for the Ki67^Medium^ group.

PgR status was adopted to define luminal B breast tumors by the 2013 St Gallen consensus, using the cut-off point of 20% proposed by Prat et al. [27]. However, this cut-off for PgR had no prognostic significance for DFS in our ER^+^/HER2^−^ cohort. Maisonneuve et al. [28]. also found that PgR < 20% was not associated with poorer outcomes for all tumors, but only for patients with Ki67-LI of 14–20% in their study cohort. ESMO emphasized the importance of laboratory quality assurance in recommending the cut-off of 20%. The cut-off point of PgR still needs more study, just as Ki67-LI does.

This study has some limitations. First, this was a single-institution retrospective study, and its results might not be directly applicable to other institutions. Before putting these results into clinical practice, other institution should verify them with their own laboratory data. In addition, the follow-up period of this cohort was relatively short considering that in the ER^+^/HER2^−^ subset, most cases of recurrence—let alone death—occur at least 5 years later [29, 30]. For this reason, we did not analyze overall survival in this study.

## Conclusions

We have evaluated distribution of Ki67-LI at an Asian institution and confirmed its validity as a risk factor among ER^+^/HER2^−^ BCs according to the 2015 St Gallen consensus. For patients with clearly very low or very high Ki67-LIs in early-stage BCs, the importance of Ki67 in clinical decisions is rather straightforward. However, for patients whose Ki67-LIs are in a medium range, diagnostic parameters including tumor size, lymph node involvement, and grade might provide significant clues.

## Additional files

Additional file 1: Table S1. Results of disease-free survival analysis by Cox proportional hazards model for the the Ki67^Low^ group. (DOC 75 kb)
Additional file 2: Table S2. Results of disease-free survival analysis by Cox proportional hazards model for the the Ki67^High^ group. (DOC 76 kb)

## Abbreviations

ALND: Axillary lymph node dissection
ASCO: American Society of Clinical Oncology
BC: Breast cancer
BCS: Breast-conserving surgery
DFS: Disease-free survival
ER+/HER2−: Estrogen receptor-positive and human epidermal growth factor receptor 2-negative
ESMO: European Society for Medical Oncology
FISH: Fluorescence in situ hybridization
IDC: Invasive ductal carcinoma
IHC: Immunohistochemical
ILC: Invasive lobular carcinoma
IQT: Interquartile range
LI: Labeling index
LVI: Lymphovascular invasion
NCCN: National Comprehensive Cancer Network
PgR: Progesterone receptor
SLNB: Sentinel lymph node biopsy
