# Supplementary material for: Prognostic significance of Ki67 in Chinese women diagnosed with ER+/HER2− breast cancers by the 2015 St. Gallen consensus classification
Source: BMC Cancer. 2017 Jan 6;17:28. doi: 10.1186/s12885-016-3021-7 (PMC5219721; doi:10.1186/s12885-016-3021-7)
Supplement: Additional file 1: Table S1. — Results of disease-free survival analysis by Cox proportional hazards model for the the Ki67Low group. (DOC 75 kb) [file 12885_2016_3021_MOESM1_ESM.doc]

**Additional file 1: Table S1**

**Results of disease-free survival analysis by Cox proportional hazards model for the the Ki67^Low^ group**

|  |  | Univariate | | |  | Multivariate | | | |
| --- | --- | --- | --- | --- | --- | --- | --- | --- | --- |
|  | HR 95% CI P value | | | |  | HR 95% CI P value | | | |
| Age (years) |  |  |  |  |  |  |  |  |  |
| 35-50 vs<35 | 4191.555 | 0.000 | 2.34E120 | 0.952 |  | 2701.464 | 0.000 | 1.31E102 | 0.946 |
| 51-65 vs<35 | 19091.269 | 0.000 | 1.06E121 | 0.943 |  | 14185.276 | 0.000 | 6.86E102 | 0.934 |
| >65 vs<35 | 18077.807 | 0.000 | 1.01E121 | 0.943 |  | 12468.972 | 0.000 | 6.06E102 | 0.935 |
| pT |  |  |  |  |  |  |  |  |  |
| T2 vs T1 | 1.315 | 0.329 | 5.260 | 0.698 |  | 1.299 | 0.274 | 6.161 | 0.742 |
| T3 vs T1 | 0.000 | 0.000 | —— | 0.987 |  | 0.001 | 0.000 | 3.54E136 | 0.965 |
| N |  |  |  |  |  |  |  |  |  |
| N1 vs N0 | 1.200 | 0.310 | 4.645 | 0.792 |  | 1.250 | 0.221 | 7.063 | 0.801 |
| LVI |  |  |  |  |  |  |  |  |  |
| positive vs negative | 3.402 | 0.862 | 13.429 | 0.080 |  | 3.841 | 0.771 | 19.129 | 0.100 |
| Grade |  |  |  |  |  |  |  |  |  |
| III vs I/II | 0.045 | 0.000 | 2957.544 | 0.583 |  | 0.000 | 0.000 | 6.734E84 | 0.935 |
| PgR(%)  <20 vs ≥20 Surgery | 1.429 | 0.302 | 6.771 | 0.653 |  | 1.000 | 0.188 | 5.302 | 1.000 |
| Mastectomy vs BCS | 0.912 | 0.257 | 3.238 | 0.887 |  | 0.611 | 0.046 | 8.049 | 0.708 |
| Chemotherapy  No vs Yes  Radiotherapy  No vs Yes |  |  |  |  |  |  |  |  |  |
|  | 1.435 | 0.367 | 5.609 | 0.604 |  | 1.145 | 0.241 | 5.441 | 0.865 |
|  |  |  |  |  |  |  |  |  |  |
|  | 0.979 | 0.253 | 3.790 | 0.976 |  | 1.266 | 0.080 | 19.991 | 0.867 |
