# Supplementary material for: Prognostic significance of Ki67 in Chinese women diagnosed with ER+/HER2− breast cancers by the 2015 St. Gallen consensus classification
Source: BMC Cancer. 2017 Jan 6;17:28. doi: 10.1186/s12885-016-3021-7 (PMC5219721; doi:10.1186/s12885-016-3021-7)
Supplement: Additional file 2: Table S2. — Results of disease-free survival analysis by Cox proportional hazards model for the the Ki67High group. (DOC 76 kb) [file 12885_2016_3021_MOESM2_ESM.doc]

**Additional file 2: Table S2** **Results of disease-free survival analysis by Cox proportional hazards model for the the Ki67^High^ group**

|  |  | Univariate | | |  | Multivariate | | | |
| --- | --- | --- | --- | --- | --- | --- | --- | --- | --- |
|  | HR 95% CI P value | | | |  | HR 95% CI P value | | | |
| Age (years) |  |  |  |  |  |  |  |  |  |
| 35-50 vs<35 | 0.358 | 0.169 | 0.756 | 0.007 |  | 0.248 | 0.113 | 0.548 | 0.001 |
| 51-65 vs<35 | 0.378 | 0.170 | 0.843 | 0.017 |  | 0.253 | 0.107 | 0.601 | 0.002 |
| >65 vs<35 | 0.088 | 0.266 | 0.058 | 0.088 |  | 0.090 | 0.014 | 0.566 | 0.010 |
| pT |  |  |  |  |  |  |  |  |  |
| T2 vs T1 | 1.618 | 0.885 | 2.957 | 0.118 |  | 1.927 | 1.014 | 3.664 | 0.045 |
| T3 vs T1 | 2.994 | 0.885 | 10.126 | 0.078 |  | 3.473 | 0.955 | 12.627 | 0.059 |
| N |  |  |  |  |  |  |  |  |  |
| N1 vs N0 | 1.368 | 0.777 | 2.410 | 0.277 |  | 1.276 | 0.571 | 2.851 | 0.552 |
| LVI |  |  |  |  |  |  |  |  |  |
| positive vs negative | 1.211 | 0.603 | 2.432 | 0.590 |  | 1.018 | 0.481 | 2.153 | 0.964 |
| Grade |  |  |  |  |  |  |  |  |  |
| III vs I/II | 1.394 | 0.796 | 2.444 | 0.245 |  | 1.402 | 0.768 | 2.559 | 0.271 |
| PgR(%)  <20 vs ≥20 Surgery |  |  |  |  |  |  |  |  |  |
|  | 1.005 | 0.557 | 1.813 | 0.987 |  | 0.933 | 0.499 | 1.748 | 0.830 |
|  |  |  |  |  |  |  |  |  |  |
| Mastectomy vs BCS | 1.611 | 0.919 | 2.825 | 0.096 |  | 1.414 | 0.567 | 3.529 | 0.458 |
| Chemotherapy  No vs Yes  Radiotherapy  No vs Yes |  |  |  |  |  |  |  |  |  |
|  | 1.272 | 0.504 | 3.208 | 0.610 |  | 2.273 | 0.747 | 6.923 | 0.148 |
|  | 1.321 | 0.711 | 2.455 | 0.379 |  | 1.422 | 0.481 | 4.204 | 0.524 |
